# Appropriate Body Mass Index Cut-Offs to Determine Thinness, Overweight and Obesity in South Asian Children in The Netherlands

**DOI:** 10.1371/journal.pone.0082822

**Published:** 2013-12-19

**Authors:** Jeroen A. de Wilde, Paula van Dommelen, Barend J. C. Middelkoop

**Affiliations:** 1 Department of Youth Health Care, Municipal Health Service The Hague (GGD Den Haag), The Hague, The Netherlands; 2 Department of Child Health, Netherlands Organisation for Applied Scientific Research TNO, Leiden, The Netherlands; 3 Department of Life Style, Netherlands Organisation for Applied Scientific Research TNO, Leiden, The Netherlands; 4 Department of Public Health and Primary Care, Leiden University Medical Center, Leiden, The Netherlands; 5 Department of Epidemiology, Municipal Health Service The Hague (GGD Den Haag), The Hague, The Netherlands; Wayne State University School of Medicine, United States of America

## Abstract

**Background:**

Asian populations have an increased risk of developing cardiometabolic disorders at a lower body mass index (BMI) than other ethnic groups. Therefore, lower adult BMI cut-offs to determine overweight and obesity are recommended to assess the associated health risks for Asian (23 and 27.5 kg/m^2^ respectively) and Asian Indian (23, 25 kg/m^2^) populations. The objective of this study was to develop BMI cut-offs for thinness, overweight, and obesity for South Asian children in the Netherlands, and to compare the BMI cut-offs and distribution with an Asian Indian reference, the WHO Child Growth Reference, and universal BMI cut-offs.

**Methods:**

A reference cohort of 546 Surinamese South Asian boys and 521 girls, born between 1974–1976 (during the pre-obesity era) with 3408 and 3267 BMI measurements respectively, was retrospectively analysed. BMI-for-age charts were created with the LMS method. BMI centile curves passing through the cut-off points of 15 (thinness), 23 (overweight), 25 and 27.5 kg/m^2^ (obesity) at 18y were drawn as cut-off levels.

**Results:**

The BMI of Surinamese South Asian children had a similar distribution to the Asian Indian reference, apart from a lower mean and less variation. The BMI distribution differed considerably from the WHO reference and universal BMI criteria. The calculated BMI cut-offs corresponding to a BMI of 15, 23, 25, and 27.5 kg/m^2^ at 18y were at the 7.1, 81.1, 89.8, and 95.5 percentile respectively in boys, and at the 2.7, 79.5, 89.2, and 95.2 percentile in girls.

**Conclusions:**

This is the first study proposing BMI cut-offs for South Asian children based on measurements from a prosperous population unaffected by the obesity epidemic. We recommend the use of these cut-offs in South Asian children in the Netherlands as these better reflect the health risks associated with thinness, overweight and obesity, and therefore may prevent the development of cardiometabolic disorders.

## Introduction

The body mass index (BMI) is generally used as a proxy for estimating the body fat percentage and associated health risks. For many years, one set of BMI cut-offs has been used in clinical practice for all ethnic groups [1]. However, over the past decade evidence has emerged that Asian populations are at an increased risk of cardiometabolic disorders at lower BMI levels than other ethnic groups, which has been attributed to a considerably higher body fat percentage [2]. For that reason, in 2004 the World Health Organization (WHO) recommended lowering the BMI cut-offs for Asian adults, for overweight from 25 to 23 kg/m^2^ and for obesity from 30 to 27.5 kg/m^2^ [3] in anticipation of the increased health risks. However, there is evidence that these cut-offs are still too high for South Asian populations which have an increased risk of cardiovascular and metabolic disease risks at an even lower BMI [4]–[7]. This led to India adopting the lower BMI cut-off points of 23 kg/m^2^ for overweight and 25 kg/m^2^ for obesity as clinical markers requiring further intervention [8].

Although cardiometabolic risks in South Asian children and adolescents are also higher at a lower BMI than in children of European descent [9]–[11], BMI cut-offs have not yet been adjusted for this population. In several studies lowered BMI cut-offs to determine overweight and obesity in South Asian children and adolescents have been proposed [12]–[15] but none of these BMI criteria have been adopted by WHO. For all ethnic groups the universal BMI-cut-offs for ages 2–18 years [16], [17], or the WHO Child Growth Standard 0–5 years [18] and the WHO Growth Reference 5–19 years [19] are still recommended.

The universal cut-offs and the WHO Growth Reference 5–19 years were based on BMI data from studies conducted in healthy, affluent populations before the obesity epidemic began. As the rates of undernutrition and overnutrition were generally low in these populations, the corresponding BMI distributions were presumed to delineate the desirable norm [20]. The universal cut-off values were created to correspond to the centiles passing the recommended adult BMI cut-offs for thinness (16, 17, and 18.5 kg/m^2^), overweight (25 kg/m^2^) and obesity (30 kg/m^2^) at 18 years of age. The WHO cut-offs were based on standard deviation (SD) criteria (−2 SD: thinness; +1 SD: overweight; +2 SD: obesity), but these cut-offs also correspond to the universal adult BMI cut-points at 18 years of age, and thus create a continuous scale from childhood to adulthood.

Although the lowering of BMI cut-offs for overweight and obesity in South Asians is supported by evidence [21], little is known about the other end of the spectrum, thinness. Presently, no expert guidelines for thinness exist, and the current cut-offs classifying thinness are merely based on supposition [22]. The varying rates of thinness between countries [23], are generally ascribed to differences in socioeconomic factors, with the exception of South Asia. Despite favourable socioeconomic conditions [21], South Asia has the highest underweight rates in the world (based on universal criteria). However, the under-five mortality is almost half that of Sub-Saharian Africa [24]. Furthermore, in affluent Surinamese South Asian children living in the Netherlands, a disproportionately high prevalence of thinness was found [25]. This is likely to be a consequence of the high body fat percentage at low BMI, resulting in many ‘underweight’ children being misclassified. The possibility that children are being wrongly determined as underweight is supported by a recent Sri Lankan study which found that most children who were classified as underweight had a normal, or even high body fat percentage [14].

The first objective of this study was to develop South Asian specific cut-offs for the determination of thinness, overweight and obesity in children 2–18 years, based on a reference population of South Asian children who lived in a developed country during a pre-obesogenic era. The second objective was to compare the BMI distribution and cut-offs with 1) recently established Asian Indian BMI references and cut-offs for children 5–18 years [13], [26], 2) the WHO Child Growth Reference [18], [27], and 3) current universal BMI cut-offs for thinness, overweight and obesity [16], [17].

## Methods

### Subjects and data collection

The city of The Hague holds the largest community of South Asians in Continental Europe, most of whom are descendents of Asian Indians who migrated between 1873 and the 1916 from the Indian states, Uttar Pradesh and Bihar, to Suriname, a former Dutch colony [28].

As part of the Dutch health surveillance programme from birth to 19y, Youth Health Care in the Netherlands routinely performs standardized health assessments that are registered in health records. Up until the early 1990's more frequent assessments were performed: between 0 and 4y of age around 15 and from the age of 4 up to 18y at least every second year. During infancy, weight was measured at every visit, but length was less frequently measured. From the age of 1 year, length/height and weight were measured during most health assessments. For this study all length/height and weight data of a reference cohort of Surinamese South Asian children born between 1974 and 1976 were extracted from the health records of Youth Health Care, together with personal data (to determine ethnicity) and information about medical conditions or medicine use. All measurements were taken by trained Youth Health Care professionals (physicians, nurses, physician assistants). Up to the age of 1.0–2.0 years, length was measured to the nearest 0.5 cm using a measuring board in supine position with legs fully extended and heels pressed against a vertical footrest, and weight was measured to the nearest 0.01 kg with a paediatric balance beam scale. From the age of 1.5–2.0 years, height was measured on bare feet with a stadiometer or height measuring tape (microtoise) to the nearest 0.1 cm or 0.5 cm, respectively, and weight in underwear was measured with a calibrated balance beam or mechanical step scale to the nearest 0.1 kg or 0.5 kg, respectively.

### Inclusion criteria

Only records of children with a Surinamese South Asian ethnicity, as determined by parental country of birth (Suriname) and a typical Surinamese South Asian surname of the parents, were selected from the Youth Health Care archives. 12 children with a disorder or medicine use known to affect growth were excluded: diabetes (n = 2), thyroid disease (n = 2), celiac disease (n = 1), cerebral palsy (n = 2), scoliosis (n = 1), prolonged use of corticosteroids (n = 1), or treated for short stature (n = 3). In addition, all measurements below the age of 2 years from preterm children were excluded from the analyses (n = 94). Preterm birth was defined as a gestational age below 36 weeks instead of 37 weeks, as South Asian babies mature one week earlier in utero than babies of Western European decent and therefore, the lowering of the criteria for preterm birth of South Asian babies has been suggested [29]–[31].

### Ethical approval

Under Dutch law (Medical Research Involving Human Subjects Act) ethical approval for this study was not required, as this study encompassed historical routinely collected data from medical records [32]. The legal guardians of all children participating in the Youth Health Care health surveillance program gave oral consent for the health surveillance data to be stored in a medical record, and the legal guardians were also informed that anonymised data could be used in future scientific research. The dataset of this study was anonymised after the initial inclusion and coding of the required variables, by removing personal data such as (family) names, country of birth and date of birth. All data analyses were performed on this dataset. The head of the department of Youth Health Care of the Municipal Health Service of the city of the Hague and other medical staff approved the study protocol and gave permission to use the data required for this study.

### Statistical analyses

To determine the BMI cut-offs for the Surinamese South Asian population, the same methodology was applied as was used to establish the universal BMI cut-offs [33]. Separate BMI-for-age charts for males and females were determined with the LMS method [34], using R statistical software (v2.14.0) and the GAMLSS package (v4.1-0). This method transforms data into a normal distribution resulting in three smoothed age-dependent curves for skewness (L), median (M) and coefficient of variation (S), that accurately describe the characteristics of the distribution, when combined. The more L deviates from a value of 1, which signifies a symmetric distribution, the stronger the distribution is skewed. The number of degrees of freedom determines the smoothness of the curves and how well they fit the data. By assessing worm plots the appropriate amount of smoothing was applied [35]. To obtain a better fit in age periods where the LMS parameters have steeper slopes, age was log-transformed. After an optimal fit was achieved, age was transformed back to the original distribution and the LMS parameters per sex and age were calculated.

As a BMI of 23 and 27.5 was proposed to define overweight and obesity in Asian populations [3] in general, and a BMI of 23 and 25 for overweight and obesity in Asian Indians [9], the standard deviation scores or Z-scores (Z) that pass a BMI of 23, 25 and 27.5 at 18 years for males and females separately were calculated with the formula:
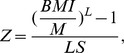
where BMI has a value of 23, 25 or 27.5 and L, M and S are the calculated values at age 18 of the population of interest. Similarly to the WHO criteria, a Z-score of −2 was initially chosen as the thinness criterion.

Based on the found Z-scores for each BMI class, the corresponding cut-offs for sex and age are calculated with the formula: 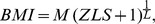
where Z is the value determined in the former step and L, M, and S the values for the specific age and sex.

To measure the agreement between the BMI classes (normal weight, overweight and obesity) determined by the BMI cut-offs of this study and by the previously determined Asian Indian cut-offs [13], Cohen's kappa was calculated with IBM SPSS Statistics v20. To compare the agreement for each BMI category separately, the BMI categories were dichotomised into three variables: normal weight versus no normal weight, overweight versus no overweight, and obesity versus no obesity.

## Results

A total of 546 boys with 3408 BMI measurements, and 521 girls with 3267 measurements were included in this study. 2746 measurements were of children aged 0–3 years and 3929 of children 4–18 years. The LMS values of the distributions are shown in table 1. At all ages the L-values were lower than 1 which indicates skewedness to the right. The coefficient of variation S was relatively low up to the age of five but progressively increased with age, signifying that the range of BMI's was increasing.

**Table 1:** L (skewness), M (median), and S (coefficient of variation) values for BMI (kg/m^2^) by age and sex of Surinamese South Asian reference cohort 1974–1976.

	Boys			Girls		
Age in years	L	M	S	L	M	S
0.0383 (2 weeks)	−0.0519	12.24	0.0919	−2.3969	12.08	0.0636
0.0833 (1 month)	0.1012	13.46	0.0942	−0.9712	13.19	0.0726
0.1667 (2 months)	0.3242	14.69	0.0944	0.0421	14.31	0.0807
0.3333 (4 months)	0.6674	15.91	0.0924	0.6035	15.50	0.0857
0.5 (6 months)	0.8338	16.47	0.0904	0.6810	16.05	0.0864
0.6667 (8 months)	0.8635	16.72	0.0887	0.6407	16.23	0.0859
0.8333 (10 months)	0.7995	16.75	0.0874	0.5453	16.23	0.0849
1	0.6865	16.65	0.0864	0.4296	16.14	0.0838
1.5	0.2852	16.11	0.0847	0.0700	15.68	0.0812
2	−0.0875	15.54	0.0843	−0.2353	15.21	0.0801
2.5	−0.4192	15.10	0.0849	−0.4932	14.88	0.0802
3	−0.7068	14.78	0.0864	−0.7255	14.64	0.0813
3.5	−0.9497	14.56	0.0886	−0.9359	14.46	0.0831
4	−1.1512	14.39	0.0912	−1.1225	14.30	0.0855
4.5	−1.3174	14.26	0.0942	−1.2791	14.17	0.0885
5	−1.4538	14.17	0.0974	−1.4026	14.09	0.0918
5.5	−1.5641	14.12	0.1008	−1.4942	14.07	0.0953
6	−1.6513	14.12	0.1044	−1.5587	14.10	0.0990
6.5	−1.7183	14.18	0.1082	−1.6014	14.19	0.1027
7	−1.7657	14.29	0.1119	−1.6266	14.33	0.1065
7.5	−1.7945	14.45	0.1158	−1.6380	14.51	0.1102
8	−1.8059	14.63	0.1196	−1.6385	14.72	0.1139
8.5	−1.8016	14.84	0.1234	−1.6303	14.96	0.1174
9	−1.7831	15.08	0.1272	−1.6148	15.22	0.1209
9.5	−1.7519	15.33	0.1310	−1.5929	15.50	0.1242
10	−1.7099	15.59	0.1347	−1.5655	15.78	0.1274
10.5	−1.6586	15.86	0.1384	−1.5338	16.07	0.1305
11	−1.5995	16.13	0.1420	−1.4988	16.37	0.1334
11.5	−1.5342	16.40	0.1455	−1.4615	16.65	0.1362
12	−1.4640	16.67	0.1490	−1.4229	16.94	0.1390
12.5	−1.3904	16.93	0.1524	−1.3839	17.22	0.1416
13	−1.3150	17.19	0.1558	−1.3450	17.50	0.1442
13.5	−1.2392	17.43	0.1591	−1.3065	17.76	0.1467
14	−1.1638	17.68	0.1624	−1.2686	18.02	0.1492
14.5	−1.0894	17.91	0.1656	−1.2316	18.28	0.1516
15	−1.0162	18.13	0.1687	−1.1957	18.52	0.1539
15.5	−0.9445	18.35	0.1718	−1.1608	18.75	0.1563
16	−0.8745	18.56	0.1749	−1.1270	18.98	0.1585
16.5	−0.8064	18.76	0.1779	−1.0941	19.20	0.1608
17	−0.7402	18.96	0.1809	−1.0623	19.42	0.1629
17.5	−0.6758	19.15	0.1839	−1.0315	19.62	0.1651
18	−0.6133	19.34	0.1868	−1.0013	19.83	0.1672

**Figure 1:**
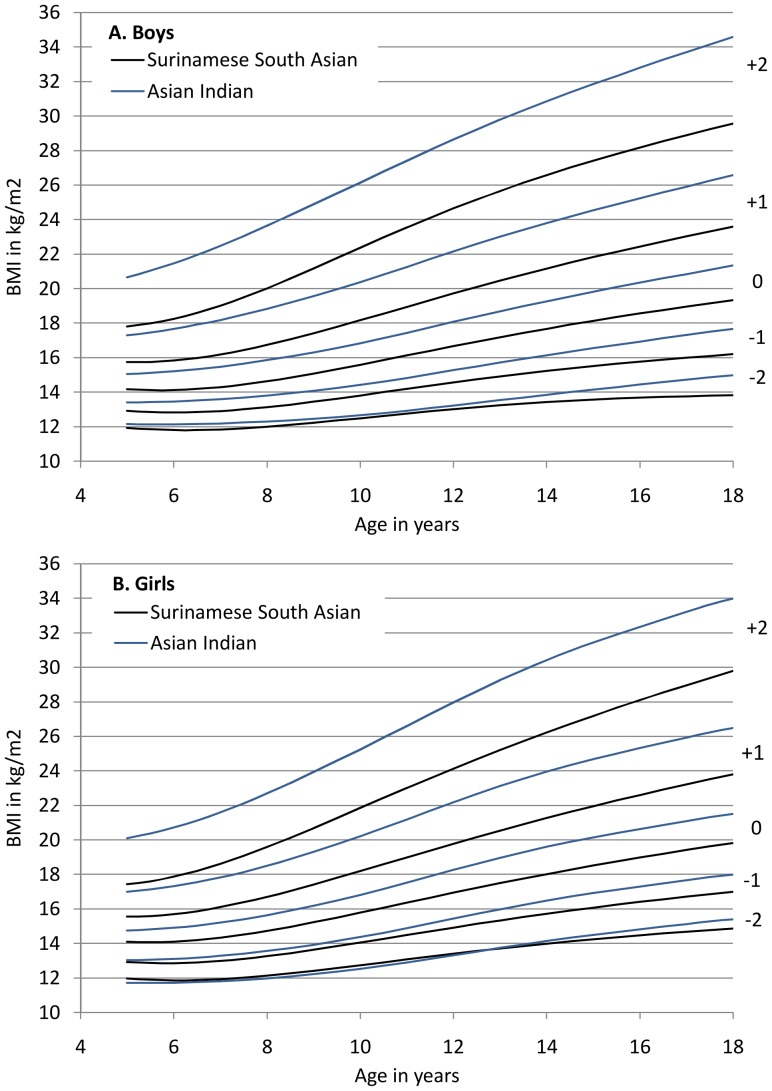
BMI distribution for ages 0–18 years of Asian Indian reference^22^ and Surinamese South Asian cohort 1974–1976, boys (A) and girls (B).

The BMI distribution was consistent with a recent Asian Indian reference 5–18 years (figure 1), showing a similar skewedness, and therefore a similar distributional pattern of BMI. Also, the lower end of the distribution coincided with that of Asian Indian children, reflecting similar thinness rates in the Indian population. However, in the Surinamese South Asian population at every age the median and coefficient of variation was smaller.

Surinamese South Asian specific BMI cut-off points for overweight and obesity were calculated to correspond to the proposed BMI cut-offs for Asian populations of 23 and 27.5 at age 18 (table 2 and 3), and additionally to the BMI equivalent of 25 at age 18 (for obesity in Asian Indian populations).

**Table 2:** Boys –South Asian BMI cut-offs for thinness, overweight and obesity by sex and age, based on Surinamese South Asian and Asian Indian populations.

	Surinamese South Asian					Asian Indian [13]	
Age in years	Thinness (−2 SD)	Thinness (BMI 15)	Overweight (BMI 23)	Obesity (BMI 25)	Obesity (BMI 27.5)	Overweight (BMI 23)	Obesity (BMI 28)
Z-score (Percentile)	−2.00 (P2.3)	−1.47 (P7.1)	0.88 (P81.1)	1.27 (P89.8)	1.70 (P95.5)	0.36 (P64.0)	1.22 (P88.8)
2	13.1	13.7	16.7	17.3	17.9	NA	NA
2.5	12.8	13.4	16.3	16.9	17.5	NA	NA
3	12.6	13.1	16.0	16.6	17.3	NA	NA
3.5	12.4	12.9	15.8	16.4	17.1	NA	NA
4	12.2	12.7	15.7	16.3	17.1	NA	NA
4.5	12.1	12.6	15.6	16.2	17.1	NA	NA
5	11.9	12.4	15.5	16.2	17.1	15.8	17.9
5.5	11.8	12.4	15.5	16.3	17.2	15.9	18.1
6	11.8	12.3	15.6	16.4	17.4	16.0	18.4
6.5	11.8	12.3	15.7	16.6	17.7	16.1	18.7
7	11.8	12.4	15.9	16.8	18.0	16.3	19.0
7.5	11.9	12.4	16.2	17.1	18.4	16.5	19.3
8	12.0	12.6	16.4	17.5	18.8	16.8	19.7
8.5	12.1	12.7	16.8	17.9	19.3	17.0	20.1
9	12.2	12.8	17.1	18.2	19.8	17.3	20.5
9.5	12.4	13.0	17.4	18.7	20.3	17.6	21.0
10	12.5	13.1	17.8	19.1	20.8	17.9	21.4
10.5	12.6	13.3	18.2	19.5	21.3	18.3	21.9
11	12.8	13.5	18.5	20.0	21.9	18.6	22.4
11.5	12.9	13.6	18.9	20.4	22.4	19.0	22.9
12	13.0	13.8	19.3	20.8	22.8	19.3	23.3
12.5	13.1	13.9	19.6	21.2	23.3	19.7	23.8
13	13.2	14.1	20.0	21.6	23.8	20.0	24.3
13.5	13.3	14.2	20.3	22.0	24.2	20.4	24.7
14	13.4	14.3	20.7	22.4	24.6	20.7	25.1
14.5	13.5	14.4	21.0	22.8	25.0	21.0	25.5
15	13.6	14.5	21.3	23.1	25.4	21.3	25.9
15.5	13.6	14.6	21.6	23.4	25.8	21.6	26.3
16	13.7	14.7	21.9	23.8	26.2	21.9	26.7
16.5	13.7	14.8	22.2	24.1	26.5	22.2	27.0
17	13.8	14.9	22.5	24.4	26.9	22.4	27.4
17.5	13.8	14.9	22.7	24.7	27.2	22.7	27.7
18	13.8	15.0	23.0	25.0	27.5	23	28.1

NA = Not available.

**Table 3:** Girls –South Asian BMI cut-offs for thinness, overweight and obesity by sex and age, based on Surinamese South Asian and Asian Indian populations.

	Surinamese South Asian					Asian Indian [13]	
Age in years	Thinness (−2 SD)	Thinness (BMI 15)	Overweight (BMI 23)	Obesity (BMI 25)	Obesity (BMI 27.5)	Overweight (BMI 23)	Obesity (BMI 28)
Z-score (Percentile)	−2.00 (P2.3)	−1.92 (P2.7)	0.83 (P79.5)	1.24 (P89.2)	1.67 (P95.2)	0.34 (P63.3)	1.24 (P89.3)
2	13.0	13.1	16.3	16.8	17.4	NA	NA
2.5	12.8	12.8	15.9	16.5	17.1	NA	NA
3	12.6	12.6	15.7	16.3	16.9	NA	NA
3.5	12.4	12.5	15.5	16.1	16.8	NA	NA
4	12.2	12.3	15.4	16.0	16.7	NA	NA
4.5	12.1	12.1	15.3	15.9	16.7	NA	NA
5	12.0	12.0	15.3	16.0	16.7	15.4	17.6
5.5	11.9	12.0	15.3	16.0	16.9	15.5	17.8
6	11.9	11.9	15.4	16.2	17.1	15.6	18.0
6.5	11.9	11.9	15.5	16.4	17.3	15.8	18.2
7	11.9	12.0	15.8	16.6	17.7	16.0	18.5
7.5	12.0	12.1	16.0	16.9	18.1	16.2	18.9
8	12.1	12.2	16.3	17.3	18.5	16.5	19.3
8.5	12.3	12.3	16.6	17.7	18.9	16.8	19.7
9	12.4	12.5	17.0	18.1	19.4	17.1	20.2
9.5	12.6	12.7	17.3	18.5	19.9	17.4	20.7
10	12.7	12.8	17.7	18.9	20.4	17.8	21.2
10.5	12.9	13.0	18.1	19.4	21.0	18.2	21.7
11	13.1	13.2	18.5	19.8	21.5	18.6	22.2
11.5	13.2	13.3	18.8	20.2	22.0	19.0	22.8
12	13.4	13.5	19.2	20.6	22.4	19.4	23.3
12.5	13.6	13.7	19.6	21.0	22.9	19.8	23.8
13	13.7	13.8	19.9	21.5	23.4	20.2	24.3
13.5	13.9	14.0	20.3	21.9	23.9	20.5	24.8
14	14.0	14.1	20.6	22.2	24.3	20.9	25.2
14.5	14.1	14.2	20.9	22.6	24.7	21.2	25.6
15	14.2	14.4	21.3	23.0	25.2	21.5	26.0
15.5	14.4	14.5	21.6	23.3	25.6	21.7	26.3
16	14.5	14.6	21.9	23.7	26.0	22.0	26.7
16.5	14.6	14.7	22.2	24.0	26.4	22.3	27.0
17	14.7	14.8	22.4	24.4	26.8	22.5	27.3
17.5	14.8	14.9	22.7	24.7	27.1	22.8	27.6
18	14.9	15.0	23.0	25.0	27.5	23.0	27.9

NA = Not available.

Despite the sample only consisting of children born before the obesity epidemic, the overweight and obesity rates based on these cut-off values were relatively high, as indicated by the percentiles. The combined prevalence of overweight and obesity was then 18.9% in boys and 20.5% in girls.

Thinness based on a Z-score of -2 resulted in a very low BMI at age 18 of 13.8 kg/m^2^ in boys. In girls, the BMI value was higher at 14.9, but still more than 2 BMI points lower than the universally used cut-off of 17. As the recommended BMI cut-offs to determine overweight and obesity in Asian adults are respectively 2.0 and 2.5 BMI points lower than universal BMI criteria, a single adult BMI equivalent of 15 kg/m^2^ was chosen as cut-off to determine thinness, which corresponds to the 7.1th percentile in boys and the 2.7th percentile in girls.

The cut-off values for overweight were similar to the recently published Asian Indian cut-offs (table 2 and 3) [13]. Up to the age of 10 years the Surinamese South Asian cut-offs for overweight were 0.1–0.4 BMI points lower than the Asian Indian values, but from that point the values were almost identical. For obesity, a similar pattern was observed in boys, however, as the Asian Indian cut-offs correspond to a BMI of 28 at 18y, instead of 27.5, it was to be expected that the cut-offs would be lower. The BMI classes normal weight (κ = 0.96), overweight (κ = 0.88), and obesity (κ = 0.82) determined by the Surinamese South Asian BMI cut-offs and the Asian Indian cut-offs were highly comparable.

**Figure 2:**
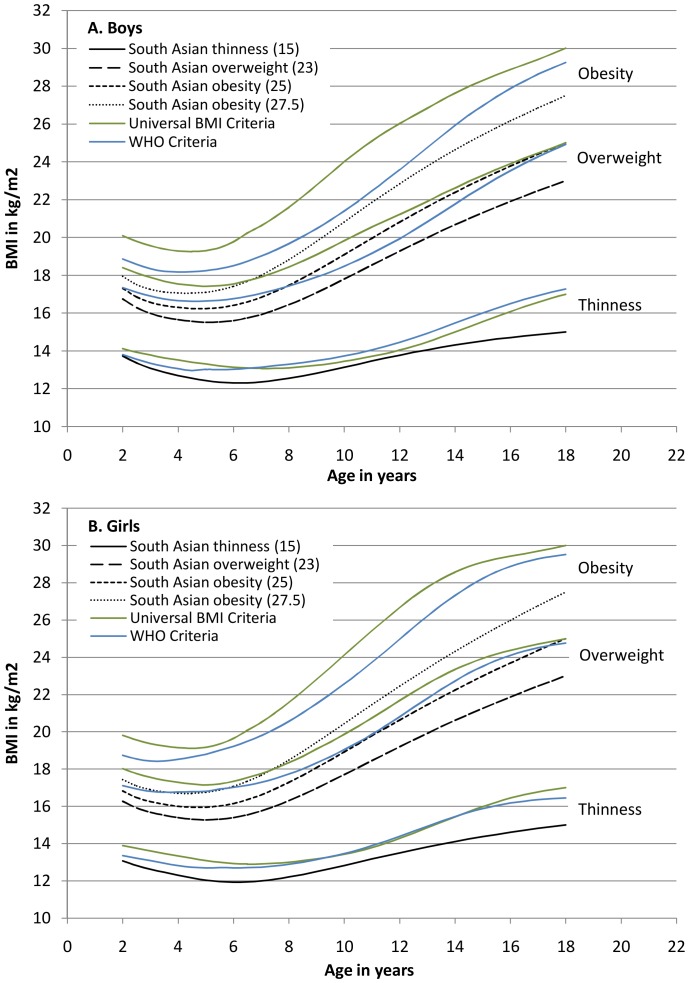
South Asian (based on adult BMI of 15, 23, 25, and 27.5), universal (BMI of 17, 25, and 30) and WHO BMI cut-offs (−2 SD, +1 SD, and +2 SD) for thinness, overweight and obesity, boys (A) and girls (B).

The centiles of the BMI distribution of the universal BMI cut-offs and of WHO differed from the Surinamese South Asian distributions (figure 2). In particular, the smaller variability of the Surinamese South Asian distribution at a young age, and the greater dispersion with increasing age, were notable. This resulted in the Surinamese South Asian obesity curve corresponding with a BMI of 27.5 at 18y being positioned below the overweight curve of the universal criteria up to the age of seven. Therefore, Surinamese South Asian children with a BMI at the higher end of the ‘normal’ range based on the universal criteria, would already be classified as obese based on ethnic specific criteria. When applying the BMI 25 criterion for obesity in South Asian children, this discrepancy became even more pronounced, as the South Asian curve stayed well below the universal overweight curve up to 18y.

## Discussion

The present universal BMI cut-offs for children insufficiently reflect the body fat percentage in South Asian children [14], [36]. Also, in this population cardiometabolic risks are higher at lower BMI thresholds compared to children of Western European decent [9], [10]. As a consequence, the assessment of the nutritional status with universal BMI criteria is inappropriate. Thus, we have developed BMI cut-offs for 2–18 year old South Asian children in the Netherlands to determine thinness, overweight and obesity, based on BMI distributions in an affluent South Asian reference population that was born before the obesity epidemic. As the overweight and obesity cut-offs correspond to the lowered adult BMI cut-offs for (South) Asian populations [3], they are expected to offer a more reliable tool for assessing the nutritional status of South Asian children, and may thus contribute to the early prevention of cardiometabolic disorders.

The BMI distributions of this reference population, and of a prosperous Asian Indian population [26], were similarly shaped, and as a result the determined BMI cut-offs for overweight and obesity based on these BMI distributions were largely similar [13]. Compared with the WHO Child Growth Reference Study [19], [37] the BMI distribution in Surinamese South Asian children differed considerably. Thinness cut-offs based on an SD of −2 resulted in very low BMI values for boys, equivalent to a value of 13.8 at age 18. As for girls the −2 SD criterions resulted in a BMI of 14.9 at this age, and as with the adult BMI cut-off for overweight in Asian populations [3], this value is 2 BMI points lower than the universal BMI cut-off.

The strengths of this study include the reliable and extensive data, the large sample size and the availability of longitudinal BMI data of a cohort born before the obesity epidemic. However, as this study encompassed cohort data, with the last measurements performed up to the early 1990's, there may have been some influence of the obesity epidemic that was likely to have started in the Netherlands as early as the late 1980's. Nevertheless, if there was an effect, it is expected to have had a minimal influence, as the children were by then early teenagers and the obesity epidemic would have a greater impact on younger children.

One of the limitations of creating BMI cut-offs based on observational data, as in this study and for the universal and WHO BMI cut-offs, is that they contain no direct information about body composition. While the centile curves used as cut-off level are linked to the established adult BMI cut levels associated with risk of morbidity at 18y, a similar body composition (including similar health risks) is assumed above the specific centile at every age in childhood. However, there may be differences in the distribution of body compositions between separate age groups. A recent study of Sri Lankan children showed that younger children had a considerably lower body fat percentage than older children [14]. If this finding is also applicable to our population, the calculated cut-offs will overestimate overweight and obesity in the younger age groups.

Waist circumference as a measure of central obesity was not available to our study, but could have had added value, considering that abdominal obesity was previously shown to be highly prevalent in urban Asian Indian children [38].

Another limitation of our set of BMI criteria is that the cut-offs were not confirmed by data of actual health outcomes. However, two recent studies that proposed adjusted BMI cut-off values for overweight and obesity in South Asian children [13], [14] also tested the validity of these cut-offs by performing metabolic panel blood tests and blood pressure measurements. In the first study the BMI cut-offs were based on body fat percentage cut-offs, which resulted in BMI values considerably lower than the values determined in our study [14]. Nevertheless, validity testing showed that the new obesity cut-offs had a higher sensitivity (37–54%) in detecting cardiometabolic risks than the universal criteria (6–11%), while the specificity was equally high (94%) [39]. The second study, the Asian Indian study with which the determined BMI cut-offs in the present study were compared, found increased cardiometabolic risks in 43–47% of the overweight children and in 72–80% of the obese children [13]. As the BMI cut-offs of this study were largely similar to the values determined in our study, the results are expected to be equally applicable to South Asian children in the Netherlands, although further research is needed to confirm this. An advantage of the present study, in contrast to the Asian Indian study, is that the cut-offs were based on historical data of healthy affluent South Asian children that were unaffected or minimally affected by the obesity epidemic. In addition, cut-offs to determine thinness, and cut-offs for children below 5 years of age were also provided.

Many children of the cohort had a low BMI, which may be due to socioeconomic factors. However, as socioeconomic indicators were not available, their relation to BMI is unknown. Nevertheless, the prevalence of childhood undernutrition in Suriname was found to be low up till 1990 [40]. Furthermore, as Surinamese migrants to the Netherlands in the 1970's had an educational level similar to the Dutch [41], the socioeconomic factors are not expected to differ from the native Dutch population. Therefore the BMI distribution of this study's population is considered representative of affluent South Asian children in the Netherlands.

It is unknown how comparable Surinamese South Asians are with South Asian populations in other countries, as mixed marriages between South Asians and other ethnic groups in Suriname, and the separation of the population for over a century, may have changed the genetic make-up. However, as Surinamese South Asians have married predominantly within their own ethnic group [28], [42], the population is expected to be fairly genetically homogenous and still comparable to other South Asian populations. The change in diet of South Asians living in Suriname may have influenced their body composition and cardiometabolic risks, but, although South Asian babies in Suriname were shown to be heavier than babies in India, a similar body composition was found [43]. The similarity in the shape of the BMI distribution of Surinamese South Asian children in our study and of Asian Indian children [13] indicates that this may also apply to older children. Moreover, as the prevalence of cardiometabolic disorders in Surinamese South Asians was shown to be at least as high [44], [45] as in South Asian populations in other countries [46]–[48], including South Asia [49], the association between body composition and risk of disease is likely to be similar to South Asians living in other countries.

In conclusion, there is convincing evidence that in South Asian children the present universal BMI cut-offs do not adequately represent the body fat percentage and associated health risks. Therefore, assessments of the nutritional status based on these BMI criteria may lead to unnecessary interventions for ‘thin’ South Asian children, whereas interventions for overweight and obesity may start at much higher BMI levels than desirable. The BMI cut-offs to determine thinness, overweight and obesity developed in the present study specifically for South Asian children living in the Netherlands, should allow more accurate assessments of their nutritional status and aid in the prevention of cardiometabolic disorders. Further research is needed to determine the ability of these new BMI criteria to predict health risks associated with a low or high BMI.
